# Intermittent Epicardial Lead Failure Detected Using a Continuous Ambulatory Electrocardiogram Monitor

**DOI:** 10.19102/icrm.2021.120601

**Published:** 2021-06-15

**Authors:** James K. Gabriels, Jose Ruiz-Morales, Ashley Tischler, Claude S. Elayi, John N. Makaryus, John N. Catanzaro

**Affiliations:** ^1^Department of Cardiology, Northwell Health, North Shore University Hospital, Manhasset, NY, USA; ^2^Department of Electrophysiology, University of Florida, Jacksonville, FL, USA; ^3^The Feinstein Institute for Medical Research, Northwell Health, Manhasset, NY, USA

**Keywords:** Continuous ambulatory electrocardiogram monitor, epicardial lead, lead failure, loss of capture

## Abstract

A 69-year-old man received epicardial pacing leads for complete atrioventricular block that occurred during a mechanical tricuspid valve replacement procedure. During follow-up, the patient reported intermittent episodes of dizziness and bradycardia. Remote transmissions and device interrogations failed to elucidate the cause of his symptoms. A continuous ambulatory electrocardiogram (ECG) monitor was used as an alternative diagnostic tool. Multiple pauses were detected by the monitor and, upon review, these events were deemed to be due to the intermittent loss of capture by the epicardial lead. Once this diagnosis was made and the malfunctioning lead was replaced, the patient’s symptoms resolved. This case highlights the novel use of a continuous ambulatory ECG monitor in diagnosing intermittent loss of capture, which was not detected by remote monitoring or device interrogations.

## Introduction

Continuous ambulatory electrocardiogram (ECG) monitoring is being used with increasing frequency in clinical practice to detect arrhythmias, assess arrhythmia burden, and monitor patients’ responses to treatment.^1–3^ More recently, mobile cardiac telemetry units have also been employed to monitor the QT interval in patients receiving QT-prolonging drugs (eg, hydroxychloroquine).^4–6^ Adhesive patch-based monitoring devices offer many advantages over traditional 24-hour Holter monitors, including greater ease of use, improved patient compliance, and extended monitoring periods.^1,7^ We report here a novel use of a continuous ambulatory ECG monitor in a pacemaker-dependent patient who was experiencing intermittent episodes of dizziness and bradycardia. Remote transmissions from his home monitor and in-office device interrogations failed to elucidate the cause of his symptoms; however, a ZIO^®^ XT Patch device (iRhythm Technologies, San Francisco, CA, USA) promptly revealed intermittent loss of capture from his epicardial lead.

## Case presentation

A 69-year-old man with a past medical history of heart failure with preserved ejection fraction, a prior mitral valve annuloplasty (32-mm annuloplasty ring; Sorin, Milan, Italy), and symptomatic sick sinus syndrome with a dual-chamber permanent pacemaker placed nine years ago presented to the hospital with lower-extremity edema. He was found to have severe tricuspid regurgitation with clinical evidence of right-sided heart failure. Due to the patient’s age and tricuspid valve anatomy, he underwent a tricuspid valve replacement with an On-X mechanical valve (On-X Life Technology, Austin, TX, USA). It was noted intraoperatively that the right ventricular (RV) pacing lead had pierced the septal leaflet of the tricuspid valve, which restricted the leaflet motion. The RV lead was surgically removed from the right ventricle and the tricuspid valve apparatus, which led to disruption of the tricuspid valve apparatus. The patient developed complete atrioventricular block intraoperatively and received two active-fixation 4046 Greatbatch^®^ Medical bipolar epicardial leads (Greatbatch Medical, Minneapolis, MN, USA), one of which was capped for redundancy. The epicardial leads were then tunneled to the existing left-sided prepectoral pocket. One of these leads was attached to his ACCOLADE model L301 permanent pacemaker (Boston Scientific, Natick, MA, USA), along with the existing transvenous atrial lead.

One week after discharge, the patient reported intermittent episodes of dizziness. He noted that his heart rate per his home blood pressure monitor was 30 bpm at times. A remote transmission from his home monitor showed that the impedance of the epicardial RV lead was 413 Ω, which was slightly lower than the implant value (500 Ω). No sensing or pacing abnormalities were noted. An in-office device interrogation for persistent symptoms revealed an increased RV pacing threshold (1.5 V at 2.0 ms vs. 0.6 V at 0.5 ms at the time of implant) and an unchanged lead impedance of 500 Ω. His underlying rhythm was sinus bradycardia with complete atrioventricular block and a ventricular escape of 30 bpm. Based on this interrogation, the device was deemed to be functioning normally. Over the course of the next month, the patient’s remote transmissions demonstrated stable atrial and ventricular lead impedances with 100% RV pacing. A 14-day ZIO^®^ XT Patch was placed because of his persistent dizziness and presyncopal spells.

**Figure 1:**
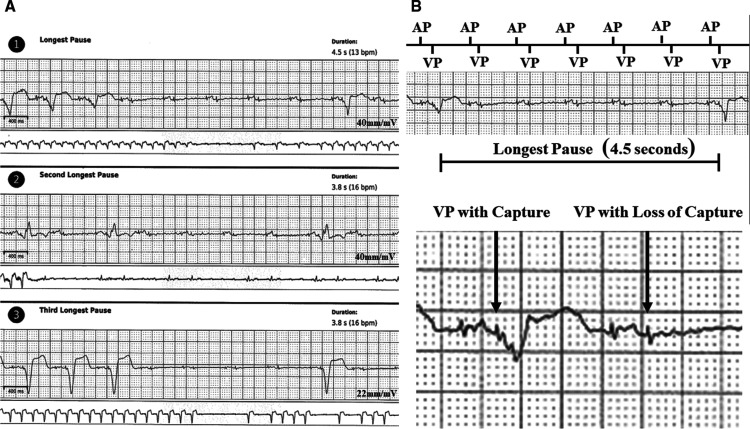
ZIO^®^ XT Patch recordings of pauses demonstrating ventricular loss of capture. **A:** Three patient-triggered events labeled as “pauses.” **B:** Further details of the longest pause (4.5 seconds). The top image indicates the presence of sequential atrial and ventricular pacing artifacts denoted by atrial and ventricular pacing. The bottom image shows an example of ventricular pacing with capture and a ventricular pacing artifact with loss of capture. AP: atrial pacing; VP: ventricular pacing.

Multiple pauses were detected by the ZIO^®^ XT Patch, the longest of which lasted for 4.5 seconds **(Figure 1)**. Review of these events revealed dual-chamber pacing with loss of capture of the ventricular lead. These pauses correlated with the patient’s symptoms as indicated by the patient’s activation of the event marker button. The patient returned the continuous ambulatory ECG monitor via mail and data were extracted in the usual fashion. Upon physician review, the patient was called and instructed to go to the emergency department. A repeat interrogation demonstrated that the RV epicardial lead threshold had risen to 3.0 V at 2.0 ms, with an unchanged level of impedance. Isometric exercises did not reproduce the failure to capture. Repeat threshold testing in a unipolar configuration revealed a similarly elevated threshold. Based on the intermittent loss of capture demonstrated by the ZIO^®^ XT Patch, the RV lead output was maximized to 6.0 V at 2.0 ms and an epicardial lead revision was planned. During the lead revision, the epicardial RV lead was found to have an impedance of more than 2,000 Ω with failure to capture. The redundant epicardial RV lead demonstrated a threshold of 1.2 V at 0.5 ms and a normal impedance. Therefore, this lead was attached to the existing generator, while the fractured ventricular lead was capped. The patient was discharged the following day. At three months of follow-up, he had not yet experienced any further symptoms or any further changes in the sensing, threshold, or impedance of his atrial or ventricular leads.

## Discussion

This case demonstrates a novel use for a continuous ambulatory ECG monitor. Our patient’s intermittent loss of capture from his epicardial lead went undiagnosed for weeks despite remote transmissions and multiple device interrogations. Use of the ZIO^®^ XT Patch quickly and accurately revealed the cause of the patient’s troubling symptoms.

Epicardial lead malfunction can occur due to elevated pacing thresholds, loss of capture, inappropriate sensing, exit block, lead displacement, fracture, or phrenic or myopotential stimulation. It is reported to occur in 27% and 44% of congenital heart disease patients with permanent epicardial pacing leads at five and 10 years, respectively.^8^ Meanwhile, lead failures attributed to fractures or dislodgements were noted in 4% of patients with steroid-eluting epicardial leads during 10 years of follow-up by Horenstein et al.^9^ Thomson et al. reported a notably higher rate of lead fractures in a study that included patients with both steroid-eluting and non–steroid-eluting leads. Moreover, they found that lead fracture was the most common cause of epicardial lead malfunction over a median follow-up of 11 years, affecting 16 of the 96 leads (16.7%) that were implanted. This group also reported that, relative to steroid-eluting leads, the use of non–steroid-eluting epicardial leads posed a significant risk for subsequent lead failure in a Cox proportional hazards model (p < 0.05).^10^ While these studies involved complications in pediatric patients with congenital heart disease,^8–10^ there are also reports of epicardial lead malfunction in adult patients.^11–13^ This relatively rare scenario most often results from damage to the lead at the time of implant or mechanical stress placed on the lead over time. In our patient’s case, the intermittent nature of the patient’s symptoms and the normal values transmitted from his remote monitor as well as the results of the in-office interrogations and fluoroscopic imaging suggest the occurrence of an acute/subacute incomplete fracture.

Irrespective of the reasoning behind the lead malfunction, the patient remained symptomatic and without a clear explanation for his symptoms for five weeks. Notably, the automatic capture feature for the RV lead was not activated in the weeks following the implant. It is possible that daily threshold testing with automatic capture, which has been demonstrated to be safe in patients with epicardial leads, may have increased the chances of detecting the malfunction sooner.^14,15^ Despite features such as automatic capture and other advances in home monitoring technology, which now allow for daily assessments of lead integrity, this modality failed to reveal that the lead was functioning abnormally. This is not unexpected given that the loss of capture was intermittent. Additionally, in-office impedance and threshold testing while the patient was performing provocative maneuvers did not sufficiently reproduce the loss of capture. Given that the loss of capture was occurring relatively infrequently, it would have been difficult to detect during an in-office interrogation unless the loss of capture happened to occur at the time the device was being tested. Based on the sporadic nature of the patient’s symptoms, a 14-day continuous ambulatory ECG monitor was prescribed, which ultimately established the correct diagnosis.

It should be noted that, although the diagnosis was made using the ZIO^®^ XT Patch in this case, there are other devices, such as mobile continuous telemetry monitors, that have similar arrhythmia detection capabilities. An important consideration when choosing an extended ambulatory monitor, especially when investigating cases involving a possible device malfunction, is the speed at which critical arrhythmias are conveyed to a clinician. In our patient’s case, given that the ZIO^®^ XT Patch does not provide real-time feedback, the provider was not made aware of the 4.5-second pause that occurred until the device had been mailed back and its data interpreted. This is a notable limitation of the ZIO^®^ XT Patch technology. Long delays between the detection of a clinically relevant arrhythmia and provider notification could have a negative impact on patient care. Mobile continuous telemetry units, such as the ZIO^®^ AT Patch (iRhythm Technologies), the MCOT Patch (BioTelemetry, Malvern, PA, USA), and the NUVANT Mobile Cardiac Telemetry (Corventis, San Jose, CA, USA), are similar to the ZIO^®^ XT Patch in their ability to detect arrhythmias. An added benefit of these devices is that they provide near real-time feedback to clinicians.^4–6,16^ It should be emphasized that, although the feedback of these devices is significantly more rapid when compared to that of the ZIO^®^ XT Patch, notification delays of three to five minutes have been reported.^5^ Had our patient’s remote transmissions or in-office interrogation demonstrated subtle changes in the pacing threshold or lead impedance, a monitor capable of providing near–real-time feedback would have been more prudent to use. In fact, if one of these devices had been used in lieu of the ZIO^®^ XT Patch, the patient might have undergone lead revision sooner. Fortunately, our patient’s loss of capture was intermittent. Had the loss of capture lead to longer periods without ventricular pacing, the delay in notification time could have been potentially life-threatening. Despite this limitation of the ZIO^®^ XT Patch, it does provide extended arrhythmia monitoring without the need for a separate portable data transmission device, which is required when using the ZIO^®^ AT Patch, the NUVANT Mobile Cardiac Telemetry monitor, and the MCOT Patch. Potential limitations of devices that require a separate portable data transmission device include a reliance on the supply of electricity, nonuniform cellular coverage, as well as a need to have the monitor in close proximity to the device in order to transmit information. Issues with usability, inability to transtelephonically download information, and patient adherence are further limitations.^17^

## Conclusions

To the best of our knowledge, this is the first report of a continuous ambulatory ECG monitor being used to diagnose intermittent epicardial lead failure. This novel use of the described monitoring device yielded the patient’s diagnosis after multiple remote transmissions and in-office device interrogations failed to elucidate the cause of his symptoms.

## References

[1] Sana F, Isselbacher EM, Singh JP, Heist EK, Pathik B, Armoundas AA (2020). Wearable devices for ambulatory cardiac monitoring: JACC state-of-the-art review. J Am Coll Cardiol.

[2] Turakhia MP, Hoang DD, Zimetbaum P (2013). Diagnostic utility of a novel leadless arrhythmia monitoring device. Am J Cardiol.

[3] Go AS, Reynolds K, Yang J (2018). Association of burden of atrial fibrillation with risk of ischemic stroke in adults with paroxysmal atrial fibrillation: the KP-RHYTHM study. JAMA Cardiol.

[4] Gabriels J, Saleh M, Chang D, Epstein LM (2020). Inpatient use of mobile continuous telemetry for COVID-19 patients treated with hydroxychloroquine and azithromycin. HeartRhythm Case Rep.

[5] Chang D, Saleh M, Gabriels J (2020). Inpatient use of ambulatory telemetry monitors for COVID-19 patients treated with hydroxychloroquine and/or azithromycin. J Am Coll Cardiol.

[6] Braunstein ED, Reynbakh O, Krummerman A, Di Biase L, Ferrick KJ (2020). Inpatient cardiac monitoring using a patch-based mobile cardiac telemetry system during the COVID-19 pandemic. J Cardiovasc Electrophysiol.

[7] Barrett PM, Komatireddy R, Haaser S (2014). Comparison of 24-hour Holter monitoring with 14-day novel adhesive patch electrocardiographic monitoring. Am J Med.

[8] Lau KC, Gaynor JW, Fuller SM, Smoots KA, Shah MJ (2015). Long-term atrial and ventricular epicardial pacemaker lead survival after cardiac operations in pediatric patients with congenital heart disease. Heart Rhythm.

[9] Horenstein MS, Hakimi M, Walters H, Karpawich PP (2003). Chronic performance of steroid-eluting epicardial leads in a growing pediatric population: a 10-year comparison. Pacing Clin Electrophysiol.

[10] Thomson JDR, Blackburn ME, Van Doorn C, Nicholls A, Watterson KG (2004). Pacing activity, patient and lead survival over 20 years of permanent epicardial pacing in children. Ann Thorac Surg.

[11] Burger H, Pecha S, Hakmi S, Opalka B, Schoenburg M, Ziegelhoeffer T (2020). Five-year follow-up of transvenous and epicardial left ventricular leads: experience with more than 1000 leads. Interact Cardiovasc Thorac Surg.

[12] Blommaert D, Deprez F, Eucher P (2002). Loss of left ventricular epicardial lead capture due to pneumothorax. Pacing Clin Electrophysiol.

[13] Chu DJ, Lam WW (2020). Epicardial unipolar lead loss of ventricular capture during radiofrequency ablation of atrial fibrillation. Case Rep Cardiol.

[14] Tomaske M, Harpes P, Pretre R, Dodge-Khatami A, Bauersfeld U (2007). Long-term experience with AutoCapture-controlled epicardial pacing in children. Europace.

[15] Bauersfeld U, Nowak B, Molinari L (1999). Low-energy epicardial pacing in children: the benefit of autocapture. Ann Thorac Surg.

[16] Engel JM, Mehta V, Fogoros R, Chavan A (2012). Study of arrhythmia prevalence in NUVANT Mobile Cardiac Telemetry system patients. Annu Int Conf Proc IEEE Eng Med Biol Soc.

[17] Zimetbaum P, Goldman A (2010). Ambulatory arrhythmia monitoring choosing the right device. Circulation.

